# The mechanism study of Miao medicine Tongfengting decoction in the treatment of gout based on network pharmacology and molecular docking

**DOI:** 10.1097/MD.0000000000032300

**Published:** 2022-12-23

**Authors:** Xin Peng, Cong Huang, Nannan Zhang, Yuepeng Cao, Zhigang Chen, Wukai Ma, Zhengqi Liu

**Affiliations:** a Guizhou University of Traditional Chinese Medicine, Guiyang, P.R. China; b Basic medical college, Guizhou University of Traditional Chinese Medicine, Guian District, Guiyang, P.R. China; c Guizhou Province Key Laboratory of Prescription and Syndrome Pharmacology in Chinese Medicine, Guian District, Guiyang, P.R. China; d Second Affiliated Hospital of Guizhou University of Traditional Chinese Medicine, Guiyang, P.R. China.

**Keywords:** gout, Miao medicine Tongfengting decoction, molecular docking, network pharmacology, potential target

## Abstract

This study sought to clarify the mechanism of action of Miao medicine Tongfengting decoction in the treatment of gout through network pharmacology and molecular docking by searching for its key targets and related pathways.

**Methods:**

The active ingredients of Miao medicine Tongfengting Decoction were obtained from the TCMSP data platform, searched the relevant databases for gout-related targets,using String and Cytoscape 3.9 to build a “compound-cross-target-disease” network diagram,performed gene ontology (GO) and Kyoto encyclopedia of genes and genomes (KEGG) enrichment analysis in the DAVID database, and performed the docking analysis using PyMoL 2.3.0 and AutoDock.

**Results:**

After screening, 298 main targets of the Miao medicine Tongfengting decoction for gout were identified. The target network is established, and the topology of protein-protein interaction (PPI) network is analyzed. The enrichment analysis of KEGG pathway showed that these targets were related to Pathways in cancer, PI3K Akt signaling pathway, MAPK signaling pathway and other pathways. Molecular docking showed that the target protein had good binding power with the main active components of the compound of Miao medicine Tongfengting Decoction.

**Conclusion:**

Miao medicine Tongfengting decoction probably regulates immune mechanism using a multi-component, multi-target, and multi-pathway strategy to reduce inflammatory response and exert its therapeutic effect on gout.

## 1. Introduction

Gout is a common inflammatory arthritis known as the “King of Diseases or King’’s Disease.” It results from a long-term purine metabolism disorder that causes an elevation in uric acid level and deposition of urate crystals around the joints. The disease is most common in adult males, with a male-to-female ratio of 10:1, and its incidence and prevalence are on the rise.^[1]^ It typically presents with local redness, swelling, heat, and pain in the joints and usually occurs in the first metatarsophalangeal joint. Arthrocentesis targeted at finding specific urate crystals is the gold standard for the diagnosis of gout.^[2]^ Currently, the treatment options in modern medicine are mostly western medicine-based for the treatment of a single target. Drugs, such as ebuxostat, benzbromarone, and allopurinol, reduce uric acid level. Other drugs, such as colchicine, glucocorticoids, and non-steroidal anti-inflammatory drugs, reduce inflammation. Analgesic and symptomatic treatment options are accompanied by certain adverse reactions, such as gastrointestinal reactions and liver and kidney function damage. Moreover, they have single therapeutic targets and are associated with easy relapse after drug withdrawal.^[3–5]^ Therefore, the search for drugs with fewer side effects is a current research focus. In recent years, traditional Chinese medicine (TCM) has gradually been acknowledged to have advantages in the treatment of gout. Research in this area is also deepening, but its therapeutic mechanism needs to be further verified.

Gout is categorized as “arthralgia” and “joint-running wind” in TCM. This disease is mostly caused by the patient’s yang exuberance, damp-heat brewing internally, plus feeling of exogenous pathogens, damp heat stasis toxin blocking joints, and poor qi and blood. Therefore, treatment should focus on heat clearance, detoxification, blood circulation promotion, and turbidity removal. Miao medicine Tongfengting decoction clears heat, detoxifies, dispels wind and dampness, promotes blood circulation, and dredges collaterals. The prescription is composed of Reineckia carnea (Andr.) kunth, Musa basjoo, Luoshiteng, Sargentodoxae caulis, gypsum, Anemarrhena spp. and other medicines. Miao medicine Tongfengting decoction is a complementary and alternative treatment method for gout. Consistent with traditional Chinese medical theory, it has been used in the undergraduate room for more than 10 years, and more than 5000 individuals have visited the clinic. In previous clinical study, Miao medicine Tongfengting decoction had a definite curative effect on the clinical symptoms of patients with gouty arthritis. Moreover, it reduced TCM syndrome scores and laboratory indicators and had no obvious adverse reactions.^[6–8]^ Further studies revealed that Miao medicine Tongfengting decoction has anti-inflammatory effects by its ability to reduce serum uric acid level and inhibit cyclooxygenase-2 (COX-2) and xanthine oxidase (XOD) activities.^[9,10]^ Currently, TCM uses a multi-component and multi-target approach; its mechanism of action in the treatment of gout remains unclear, requiring further clarification.

Network pharmacology is an emerging research method that explores the relationship between drugs and diseases through active ingredients, diseases, and targets of TCM. Based on these, the molecular mechanism of drug treatment of diseases is explored. Its holistic system concept is consistent with the holistic view of TCM and principles of syndrome differentiation and treatment.^[11]^ Therefore, based on network pharmacology analysis and molecular docking technology, this study explored the molecular mechanism of Miao medicine Tongfengting decoction in the treatment of gout and provided a theoretical basis for later clinical application and novel ideas for further studies.

## 2. Materials and Methods

### 2.1. Acquisition and screening of chemical constituents of TCM in Miao medicine Tongfengting decoction

The chemical components of the Miao medicine Tongfengting decoction were obtained from the TCMSP database. The screening criteria for active pharmaceutical ingredients and protein targets were drugs with bioavailability (OB) ≥ 30% and drug similarity (DL) ≥ 0.18. Using the Uniprot Protein Database, the species parameter was set as that of humans and protein targets were normalized as gene names.

### 2.2. Collection of disease target information and screening of cross-targets

“Gout” and “gout arthritis” were used as search words to find confirmed gout-related genes on GeneCards, TTD, and OMIM human gene platforms. The Excel format of the 3 databases were downloaded and then merged together after unique gene symbol. Additionally, the Bioinformatics platform was used to obtain the potential targets of Miao medicine Tongfengting decoction in the treatment of gout, as well as the cross targets of disease and drugs.

### 2.3. Construction of protein interactions

The target of the intersection between Miao medicine Tongfengting decoction and gout were imported into the string database. The species was set to “homo sapiens,” and the minimum interaction score correlation was set to “highest confidence” (≥0.400). The drug-disease PPI network diagram was obtained initially. The TSV file was downloaded and processed using the Cytoscape 3.90 software. The protein interaction network diagram was obtained, and the core targets were filtered by analyzing parameters, such as network degree, using the Network Analyzer plug-in.

### 2.4. Construction of the network model

The compounds of the Miao medicine Tongfengting Decoction and their targets were classified. The active ingredient of the Miao medicine Tongfengting Decoction-target network for the treatment of gout was constructed using the Cytoscape 3.90 software. Using the built-in tools of the software (degree), betweenness centrality and other network topology parameters were used to screen the key active ingredients of the drug.

### 2.5. Gene ontology (GO) and Kyoto encyclopedia of genes and genomes (KEGG) pathway enrichment analyses

The shared core targets were imported into the gene function annotation analysis platform, Metascape. The species was set as the human species, and the KEGG pathway and GO enrichment analyses were submitted to establish a “core target-biological pathway” network to screen out the main pathways. The KEGG enrichment bubble chart and GO term enrichment biological process, cellular compartment, and molecular function 3-in-one histogram were generated online. The results of the analysis by Bioinformatics were used to reflect the interaction between drug targets and gout disease target-related pathways.

### 2.6. Molecular docking

The 2-dimensional structure of the key compounds were downloaded from the PubChem database. The OpenBabel software was used to convert the pdf structural formula of the compound into Mol2 format. Using the String online platform, the best structural formula of the protein was found. Thereafter, the 3-dimensional structural formula was downloaded from the PDB database. The PyMol software was used to convert the water molecules and compounds of the protein. Finally, the structural formulas of key compounds and proteins are imported into AutoDOck software for docking.

## 3. Results

### 3.1. Acquisition and screening of chemical constituents of the Miao medicine Tongfengting decoction

The names of the constituents (including Reineckia carnea (Andr.) kunth, Musa Basjoo, Luoshiteng, Sargentodoxae caulis, gypsum, Anemarrhena spp, Phellodendri chinesis, smilax tuber, coix seed) were entered into the TCMSP database. Pharmacokinetic parameters with OB ≥ 30% and DL ≥ 0.18 were set as screening conditions, and a search for the active pharmaceutical ingredients was conducted. No active ingredients were obtained from gypsum, Musa basjoo, and Reineckia carnea (Andr.) kunth on the platform. A total of 183 active ingredients (Table S1, http://links.lww.com/MD/I131, Supplemental Content, illustrate Basic information of 183 active compounds from TCMSP) and 3102 targets were identified for the remaining components. Using the Uniprot Protein Database to transform gene names to obtain 410 target genes and exclude the active ingredients that had no targets, 142 active ingredients were obtained. According to literature,^[12–17]^ gypsum, Musa Basjoo, and Reineckia carnea (Andr.) kunth have 26 active ingredients (see Table 1). The structural formulae of the active pharmaceutical ingredients were obtained from the PubChem database. Obtain compound related targets with SwissTargetPrediction database. After removing duplicates, 174 active ingredients and 1036 targets were identified for Miao medicine Tongfengting decoction.

**Table 1:** Basic information of 32 active compounds from literature.

NO.	Compound
1	4-hydroxy-3-methoxybenzaldehyde
2	dibutyl phthalate
3	1,2-Benzenedicarboxylic Acid
4	ethyl ferulate
5	vanillic acid
6	leonuriside A
7	adenosine
8	uridine
9	(4E, 6E) -1, 7-bis(4-hydroxy-3-methoxyphenyl)hepta-4, 6-dien-3-one
10	irenolone
11	bis-demethoxycurcumin
12	rel-(3S, 4aR, 10bR) -8-hydroxy-3-(4-hydroxyphenyl) -9-methoxy-4a, 5, 6, 10b-tetrahydro-3H-naphtho [2, 40-b] pyran
13	bis-(27-ethylhexyl) terephthalate
14	daucosterol
15	cyclomusalenone
16	1— methyl — cyclopentene
17	leonuriside A
18	2— hydroxy — 9 — phenyl — phenalen — 75 — one
19	2 — hydroxy — 1H — phenalen — 31 — one
20	n-Tritriacontanol
21	BETA-sitosterol
22	Daidzin
23	6-Hydroxy-3-Pyridinecarboxylic Acid
24	lupeol
25	rutin
26	3-hydroxy- oleanene
27	ferulaic acid
28	methyl betulinate
29	diosgenin
30	quercetin
31	hexacosanoic acid
32	stigmasterol

### 3.2. Collection of disease target information and screening of cross-targets

**Figure 1.**
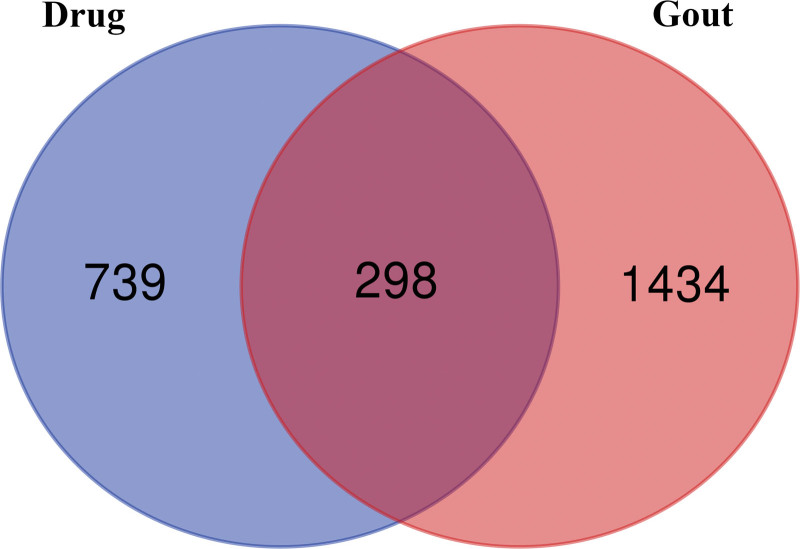
Intersection targets of Miao medicine Tongfengting decoction and gout. Note: pink represents the number of targets for gout or gout arthritis, blue represents the number of targets for active constituents of Miao medicine Tongfengting decoction, and the middle part represents the intersection targets of the 2.

A total of 1732 targets related to gout were obtained from the GeneCards database. Additionally, 18 and 10 targets were obtained from the TTD and OMIM databases, respectively. After removing duplicates, 1732 targets were identified. The disease and drug targets were imported into the Bioinformatics platform to obtain a Venn diagram, as shown in Figure 1. A total of 298 cross-targets between Miao medicine Tongfengting Decoction and gout were identified (Table 2).

**Table 2:** Common target of Miao medicine Tongfengting decoction and gout.

Prescription name	count	Target name
Miao Medicine Tongfengting Decoction	298	MMP2, XDH, HSPB1 , NTRK1 , MMP7 , EP300 , NOS2 , CCNB1 , FGR , FGFR1 , MET, MTNR1B , PTPN22 , BRCA1 , CYP2D6 , S1PR2 , CYP2C19, UGT2B7 , MAOA , ALDH2, HDAC7, GRB2 , PTK2, DNM1 , BCL2, HCRTR2, PIK3CB, SLC22A6, GCK , THRB, ESRRA , TACR1, FGFR3, PTGES, CRP , HSPA8, RARB , GSTP1 , AHR, TPMT, NFE2L2 , NR1I3, GAA , BTK , HCK , BRD4, GC, PDGFRB, CTSB, TNF, EGF, IL1A , FYN , HCRTR1 , ITGB1, LDLR, SGK1, SELP, KIT, JAK2, TNFRSF1A, CASP1 , SPP1 , F2 , XIAP, PTGS2 , ADH1C , CYP2C9, RUNX1T1, TYMS , HRAS , FOLR2, VEGFA , SLC7A11, MYC, CCNA2 , BLK, AMPD3, MMP1, STAT1 , MMP12 , SLC2A1, PTGER4 , RPS6KA2, TRPV1, PTPRC , PPARD, HMGCR , IDO1, HMOX1, MC4R , ERAP2, MMP3, CRHR1, MMP8 , GLRA1, GSTM1 , IL10 , MAPK1, ALOX5AP, FTO, CYP27B1 , MMP14 , PTPRF , CXCL2, AKT2, EGFR, SOD1 , ERBB2, IL4, ENPEP , G6PD, MAPK8, ABL1 , GCKR, FBP1 , TERT, CETP , PTPN6 , SRC , RUNX2 , PKIA, CXCL10 , TP53, MAP4K2, CDKN1A, CAT , RASA1 , ADAM17, NCOA2, PIK3R1, PDGFRA, ADRB3, SLC22A12, RORC, IDH1, SIRT1, ABCC1, IL1B , REN , NFKBIA, ALB, NOD2, AKR1A1 , P2RX7, MMP10, CTRB1, CSNK2A1, BRAF, SLC16A1, ITGAL, ALPL , COMT, SLC5A4 , SLC6A3, PNP , S1PR3 , ZAP70, PLAU , MMP13, PTK2B, NLRP3, NR1H3, EPHA2, PTPN11, PTGDR , PLCG1, DPP4 , DRD5, EZH2 , PON1, MUC1, MAP3K14, BDKRB2, B4GALT1 , ADCY5 , APOB , PPARG, MERTK , RHOA , MAPT , CXCL8 , HDAC9 , SELE, ABCB1, PIM1, THBD, MTOR , MAPK14, CSK , RAF1 , CCR2 , PLA2G4A , MPO, ITGAV , FGFR2 , HLA-A , ADAMTS4 , LDHA , SCD , CHUK , PDPK1, STAT3 , TBXAS1, ITPR1, MMEL1, SLC10A2, FFAR1 , ACHE PLAA , MCL1, CCL2, ADA, EIF2AK3, IL6 , CPT2 , HSP90AA1, VDR , ACE , PRKCE, KDR, ABCG2, EPHA3, MEN1 , RARA, PPARA, TTR, CYP1A2, LYN , SLC28A2, RBP4, IGF2 , NOD1, P2RY6, FABP1, PIK3CG, NQO1, S1PR1 , PIK3CA, FLT3 , KDM4C , IL2 , SYK , IFNG, HSPA5, TOP2A, IGF1R, ICAM1, BCL2L1, HPRT1, MAPK3 , CTSL, IL6ST , RELA , SLC2A4 , MAP2 , NOS3 , ANPEP, ADH1A, CFTR , OPRM1 , TLR4 , SERPINE1, VCAM1, INSR , PDK1 , HAAO, CDK5, JAK3 , JAK1 FABP4, AKT1 , CYP2B6 , SLC5A2, MIF , MAP2K1, ITGB3 , SAE1 , AKR1C3, PTGS1 , F3 , NR1I2, PYGM , CSF1R, JUN, IRAK4 , ADAMTS5 , ATIC , AR, CYP24A1, PRKCA , CD40LG , CYP3A4 , TUBB1, APP , RPS6KB1 , MMP9 , ADIPOQ

### 3.3. Construction of protein interactions

**Figure 2.**
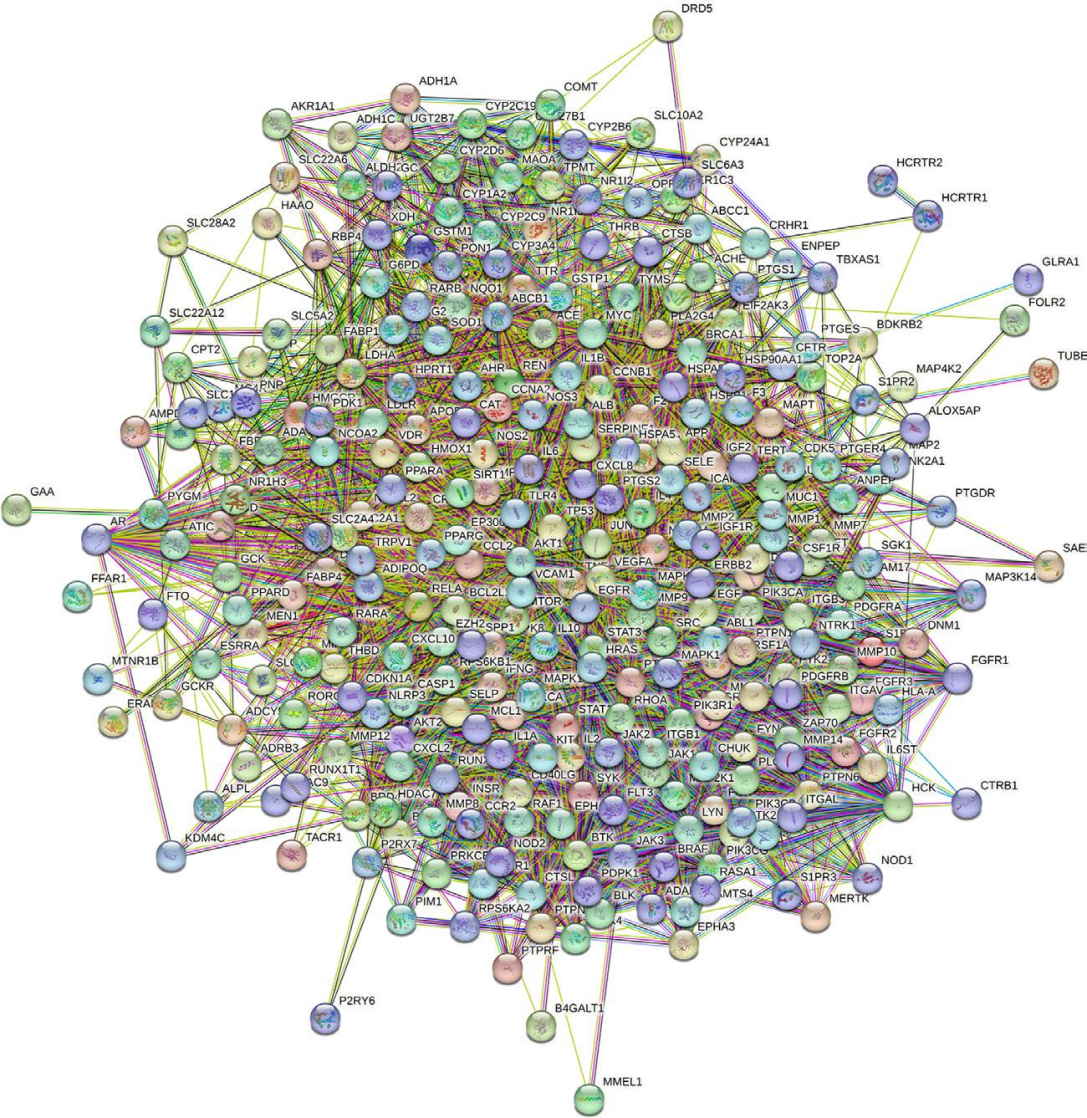
Miao medicine Tongfengting decoction and gout have a common target PPI network. The circle represents the disease target, and the line represents the relationship between the targets.

**Figure 3.**
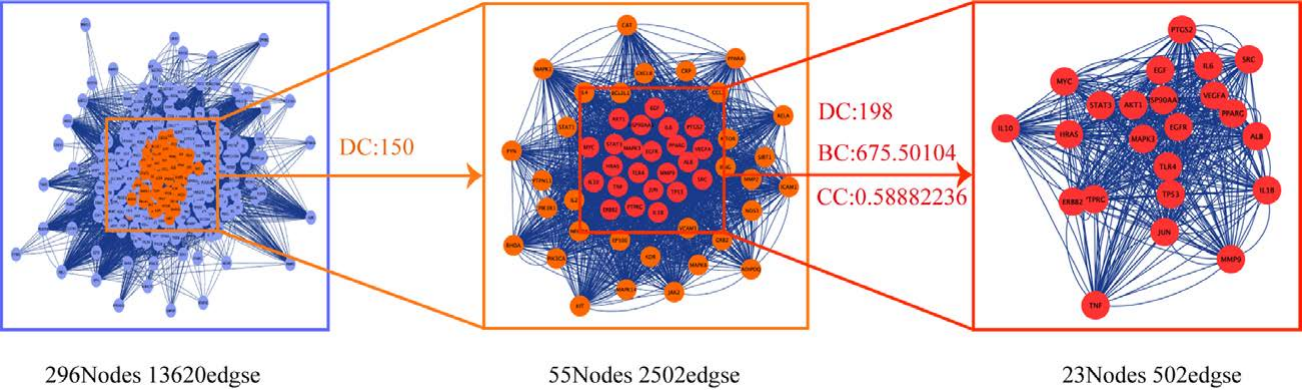
Core targets topology. The figure shows the topology of the PPI network. The color of the targets changes from Purple to red according to the DC value, and red represents the core targets. The Purple box shows all the targets, the Yellow box shows the targets whose DC value is greater than 2 times the median, and the red box shows the targets whose DC value is greater than DC:198 and BC:675.50104,CC;0.58882236, which are the core targets of this study. PPI = protein-protein interaction.

The 298 cross-targets identified between Miao medicine Tongfengting decoction and gout were imported into the String platform. After setting the corresponding parameters, the PPI network diagram was initially obtained (Fig. 2). Thereafter, the downloaded TSV file was opened with the Cytoscape 3.90 software to perform cluster analysis of the PPI network. The Network Analyzer of the platform was used to further analyze and extract targets with Degree Centrality (DC) values greater than 2 times the median or greater than the median of the 3 parameters of Degree Centrality, Closeness Centrality, and Betweenness Centrality targets. Through topology analysis extraction, 23 key targets were finally screened (See Table 3, Fig. 3).

**Table 3:** The degree value of 23 target proteins.

NO.	Gene symbol	Degree	NO.	Gene symbol	Degree
1	AKT1	362	13	MAPK3	278
2	TNF	356	14	MYC	276
3	ALB	346	15	HSP90AA1	276
4	IL6	342	16	PPARG	256
5	TP53	332	17	HRAS	252
6	VEGFA	316	18	TLR4	242
7	EGFR	310	19	PTGS2	236
8	SRC	306	20	IL10	234
9	JUN	300	21	MMP9	230
10	STAT3	298	22	ERBB2	226
11	IL1B	296	23	PTPRC	212
12	EGF	284			

### 3.4. Compounds of Miao medicine Tongfengting decoction-cross-target-disease network

**Figure 4.**
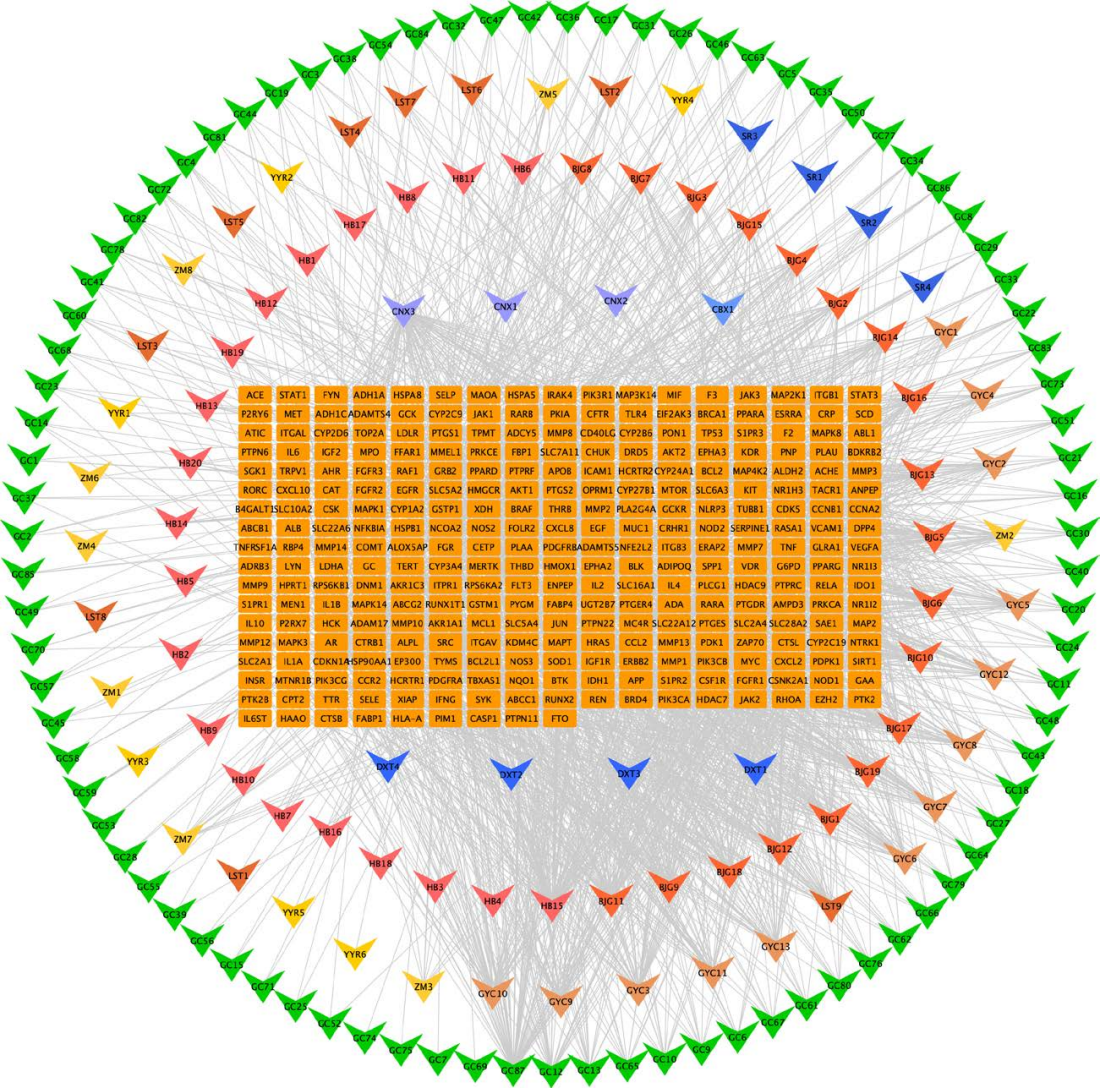
Compounds of Miao medicine Tongfengting Decoction-Cross-Target-Disease Network. Note: the orange rectangle nodes represent the intersection targets of the gout and Miao medicine Tongfengting Decoction. Other color nodes represent active medicinal ingredients.

The Cytoscape software was used to construct a network of compounds and related targets obtained from the Miao medicine Tongfengting decoction, as well as their intersecting targets with diseases (Fig. 4). The network consisted of 472 nodes (298 intersection points and 174 active ingredient points) and 2058 edges. The compounds with DC values ≥ 41 are quercetin, bis-demethoxycurcumin, ferulaic acid, (4E, 6E) -1, 7-bis(4-hydroxy-3-methoxyphenyl)hepta-4, 6-dien-3-one, 2— hydroxy — 9 — phenyl — phenalen — 75 — one, 3-hydroxy- oleanene (Table 4).

**Table 4:**
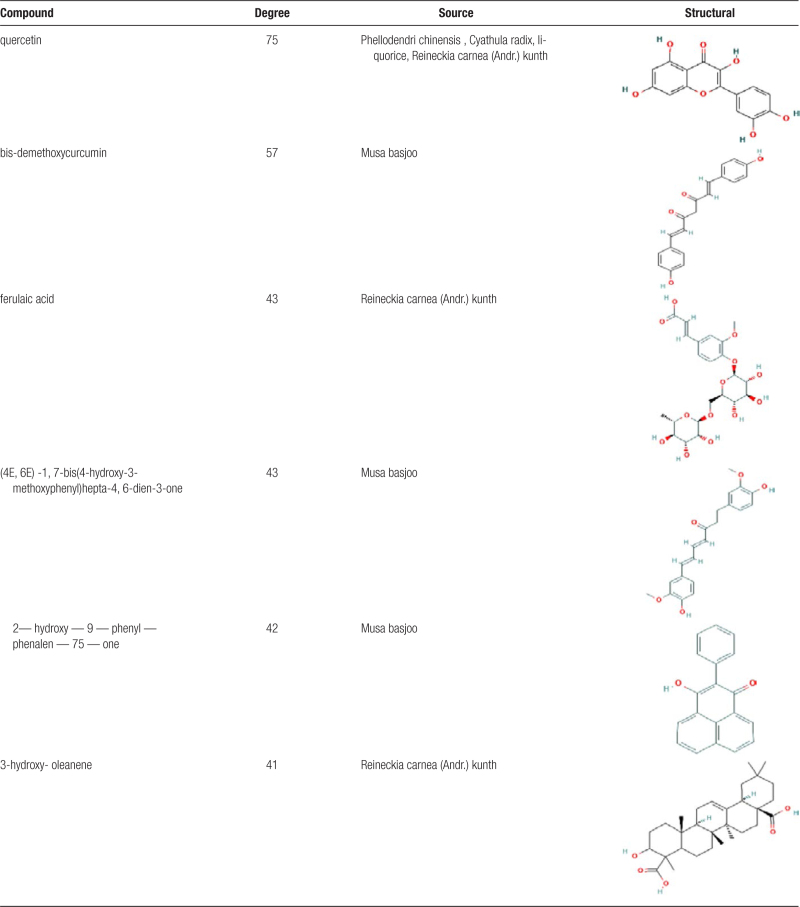
Basic information of key compounds.

### 3.5. GO and KEGG pathway enrichment analyses

**Figure 5.**
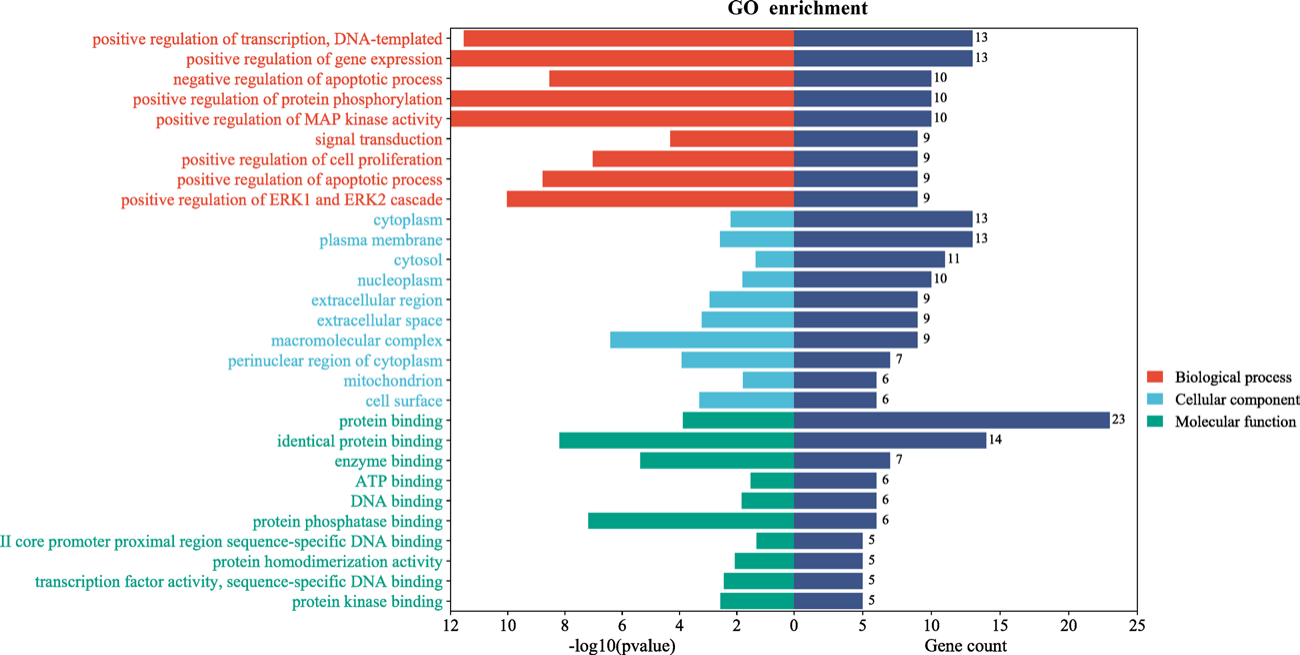
GO enrichment analysis of key targets. Note: red represents biological processes, blue represents cellular component, and green represents molecular function. GO = gene ontology.

Based on the DAVID data platform, GO and KEGG enrichment analyses of 23 common core targets were performed. GO analysis obtained 392 related biological items. *P* ≤ .05, sorted by the number of target sites. The Weishengxin online platform was used to draw the top 20 items (Fig. 5). According to Figure 5, the biological process involved in these core targets included positive regulation of transcription from RNA polymerase II promoter, positive regulation of transcription, DNA-templated, positive regulation of MAP kinase activity; cell component involved plasma membrane, cytosol, nucleoplasm; molecular function involved protein binding, identical protein binding, enzyme binding.

**Figure 6.**
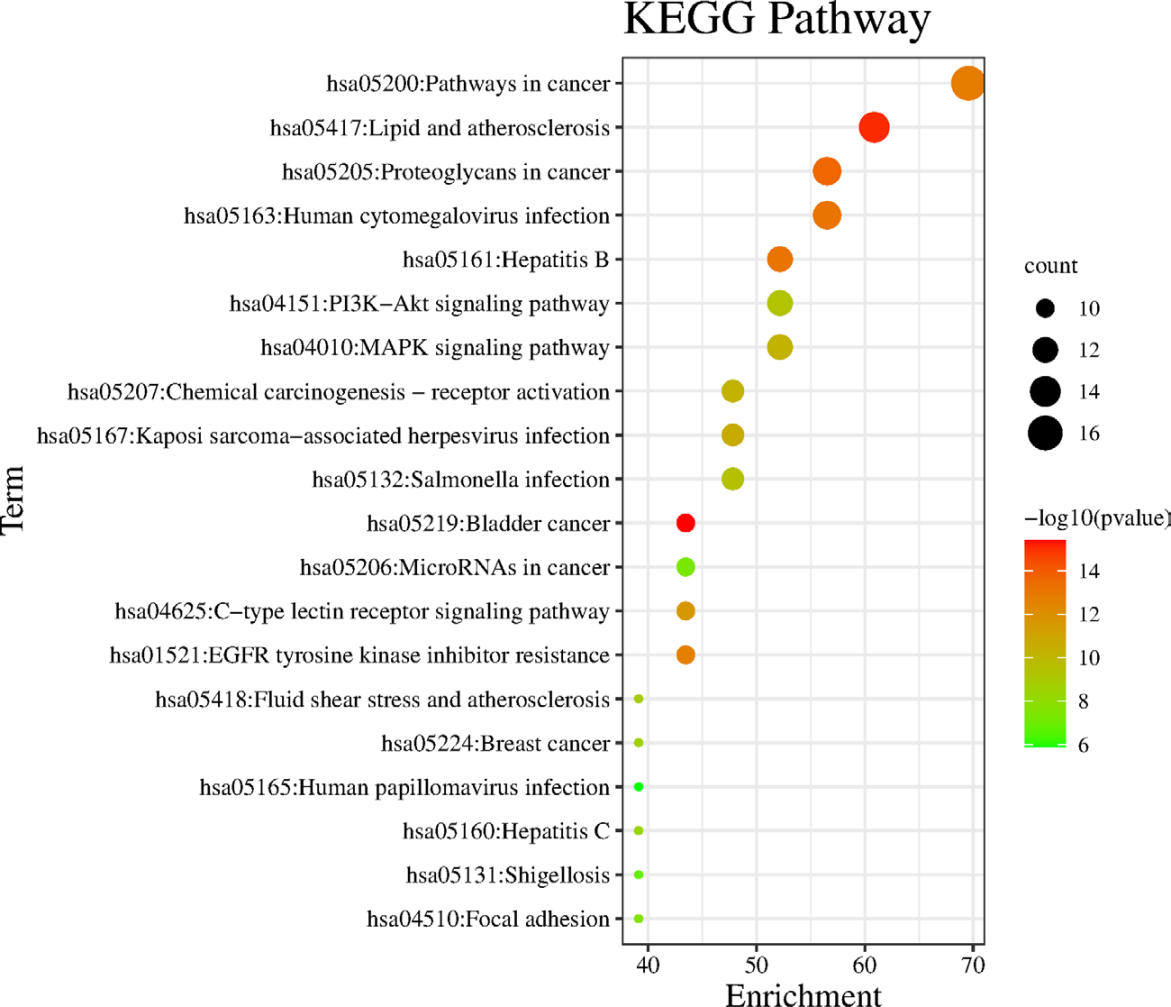
KEGG enrichment analysis of key targets Note: the x-axis represents the gene ratio, the y-axis represents the enriched pathways, the size of the dots indicates the gene number, and the color of the dots represents the *P* value. KEGG = Kyoto encyclopedia of genes and genomes.

Figure 6 shows the bubble plot results of KEGG enrichment analysis of 23 core targets. The top 20 signaling pathways were screened for analysis according to their enrichment levels (Table 5). The results show that the signaling pathways involved in the 23 core targets included the Pathways in cancer, Lipid and atherosclerosis, Human cytomegalovirus infection, MAPK signaling pathway, PI3K-Akt signaling pathway.The results suggest that these pathways may be important signal transduction in the treatment of gout by Miao Gout Stopping Soup.

**Table 5:** Target proteins in 20 signaling pathways enrichment related to gout.

KEGG ID	Description	count
hsa05200	Pathways in cancer	16
hsa05417	Lipid and atherosclerosis	14
hsa05163	Human cytomegalovirus infection	13
hsa05205	Proteoglycans in cancer	13
hsa04151	PI3K-Akt signaling pathway	12
hsa04010	MAPK signaling pathway	12
hsa05161	Hepatitis B	12
hsa05132	Salmonella infection	11
hsa05207	Chemical carcinogenesis - receptor activation	11
hsa05167	Kaposi sarcoma-associated herpesvirus infection	11
hsa05206	MicroRNAs in cancer	10
hsa04625	C-type lectin receptor signaling pathway	10
hsa01521	EGFR tyrosine kinase inhibitor resistance	10
hsa05219	Bladder cancer	10
hsa05165	Human papillomavirus infection	9
hsa05131	Shigellosis	9
hsa04510	Focal adhesion	9
hsa05160	Hepatitis C	9
hsa05224	Breast cancer	9
hsa05418	Fluid shear stress and atherosclerosis	9

### 3.6. Molecular docking results

**Figure 7.**
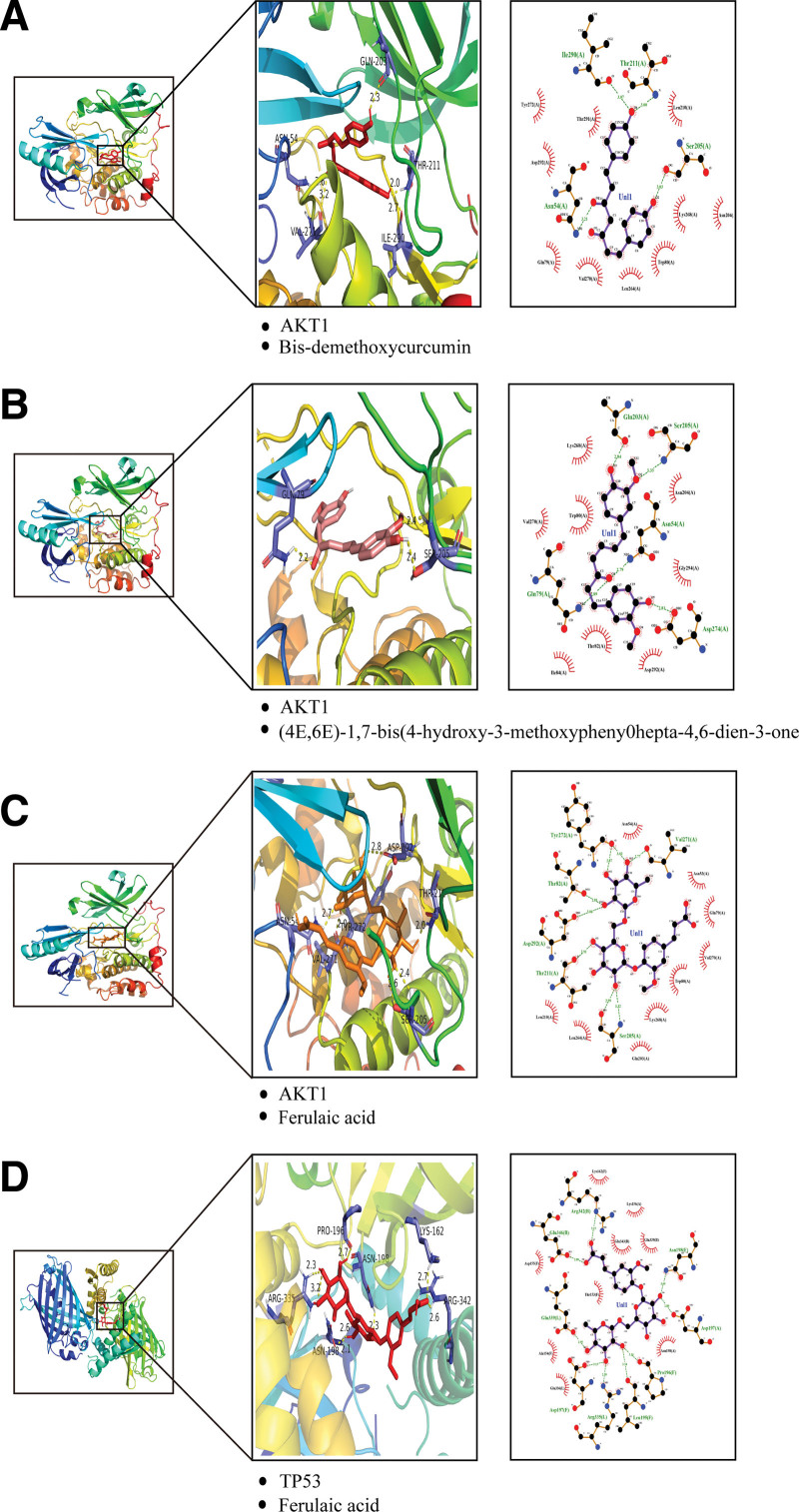
Core compound-core target molecular docking diagram. (A) Molecular docking diagram of AKT1 and bis-demethoxycurcumin; (B) Molecular docking diagram of AKT1 and (4E, 6E) -1, 7-bis(4-hydroxy-3-methoxyphenyl)hepta-4, 6-dien-3-one; (C) Molecular docking diagram of AKT1 and ferulaic acid; (D) Molecular docking diagram of TP53 and ferulaic acid.

Among the 23 core targets, the 5 target proteins with the highest Degree values, TNF, IL6, AKT1, TP53, VEGFA, were selected for molecular docking with the key pharmacodynamic molecules quercetin, bis-demethoxycurcumin, ferulaic acid, (4E, 6E) -1, 7-bis(4-hydroxy-3-methoxyphenyl)hepta-4, 6-dien-3-one,and the results are detailed in Table 6. The absolute values of the docking scores indicate the affinity of the components for the target and the stability of the conformation. Absolute values greater than 4.25 indicate specific binding activity, greater than 5.0 indicate good binding activity; greater than 7.0 indicates strong binding activity.^[18]^ According to the absolute value after binding indicated a good binding activity between the key active ingredient and the key target, which further proved the therapeutic effect of Miao Gout Stopping Soup on gout. The molecules with strong binding activity were selected for docking demonstration, as shown in Figure 7.

**Table 6:** Docking results of core target proteins and core active components.

Compound	TNF	IL6	TP53	VEGFA	AKT1
(4E, 6E) -1, 7-bis(4-hydroxy-3-methoxyphenyl)hepta-4, 6-dien-3-one	-7.2	-7.1	-8	-6.4	-9.7
quercetin	-8.1	-7.7	-8.8	-7.1	-9.4
bis-demethoxycurcumin	-7.1	-6.4	-7.9	-7.1	-9.7
ferulaic acid	-8.6	-7.6	-9.5	-8.2	-10.1

## 4. Discussion

Previous clinical and experimental studies verified Miao medicine Tongfengting decoction has a significant effect on the treatment of gouty arthritis. This compound can significantly improve the clinical symptoms of patients, including pain relief. However, its mechanism of action remains unclear. Therefore, through network pharmacology and molecular docking technology, this study explored the molecular mechanism of Miao medicine Tongfengting decoction in the treatment of gouty arthritis to provide a good theoretical basis for clinical treatment.

Gout is more of an autoinflammatory disease than a purely metabolic disease.^[19]^ It is caused by hyperuricemia or persistently elevated serum uric acid levels that promote the deposition of urate crystals in the joint cavity, resulting in inflammatory arthritis.^[20]^ The disease is characterized by recurrent episodes in the acute phase. Gout presents as redness, swelling, heat, and pain in the affected joints with limited joint mobility. NLRP3, a member of the NOD-like receptor (NLR) family, is a multimeric protein. It plays an important role in innate and adaptive immune responses, evidenced by its contribution to autoimmune diseases, such as rheumatoid arthritis, systemic lupus erythematosus, and Sjögren’s syndrome.^[21]^ NLRs are the main regulators of immune response. When the NLPR3 inflammasome is overactivated, it induces the activation of caspase-1, resulting in the high expression and secretion of IL-1β and IL-18 into the extracellular space. The secretion aggravates the inflammatory response and increases the degree of joint redness, swelling, heat, and pain.^[22]^ In addition, Toll-like receptors (TLRs) play an important role in the regulation of the immune system and are involved in the process of inflammation and apoptosis.^[23]^ The TLR family recognizes specific signatures of invading microbial pathogens and danger-associated molecules. Its signaling pathway is divided into the MyD88-dependent pathway and TRIF-dependent pathway. The receptor activates downstream inflammatory cytokine production through signaling.^[24]^ Urate crystals, as danger signaling molecules, induce activation of the TLR4 receptor when it is recognized by TLR4 and interact with the MyD88 adapter to promote the activation of signaling cascades, such as NF-KB and MAKP. Moreover, they produce pro-inflammatory cytokines, such as IL-1, IL-6, and TNF-α; induce NLRP3 inflammasome expression; and release more inflammatory mediators.^[22,25]^ Miao medicine Tongfengting decoction dispels pathogens and detoxifies, promoting blood circulation, removing turbidity, dispelling wind, and removing dampness. In preliminary clinical studies, Miao medicine Tongfengting decoction was found to effectively relieve joint pain, reduce inflammatory indicators, and significantly reduce the expression of TLR2/TLR4/MyD88 and other proteins in the joint synovium of gouty arthritis rats, reducing the inflammatory response.^[26,27]^

Through network topology parameter screening, the key targets of Miaoyao Tongfengting Decoction in the treatment of gout are AKT1, TNF, ALB, IL6, TP53, VEGFA, EGFR, SRC, JUN, STAT3, IL1B, EGF, MAPK3, MYC, HSP90AA1, PPARG, HRAS, TLR4, PTGS2, IL10, MMP9, ERBB2, PTPRC. The target with the highest Degree value was selected for molecular docking verification, and the results showed that the active ingredient could bind well to the target. AKT1 has the highest degree score in PPI and the strongest binding ability to key compounds, and is considered to be the pivotal target of Miaoyao Tongfengting decoction in the treatment of gout. AKT1 is a serine/threonine protein kinase and is a member of the AKT family. At present, 3 different isoforms of the AKT family have been found, namely AKT1, AKT2 and AKT3. AKT has been shown to play a key role in tumor cell proliferation, apoptosis, and protein synthesis. In addition, current studies have found that this target also plays an important role in the development and function of innate immune cells, and essential for the regulation of inflammatory responses.^[28]^ AKT can regulate the secretion of inflammatory cytokines in neutrophils, and TLR2 is a receptor for Gram-positive bacteria-derived peptidoglycan and lipoproteins, which can be expressed in neutrophils. In the signal transduction pathway of TLR2, Activated AKT results in the rapid release of proinflammatory cytokines and chemokines.^[29,30]^ That is to say, activated AKT participates in inflammatory response by phosphorylating various downstream signaling molecules in neutrophils.

According to the DC value of the main active ingredients, quercetin, bis-demethoxycurcumin, ferulaic acid, (4E, 6E) -1, 7-bis(4-hydroxy-3-methoxyphenyl)hepta-4, 6-dien-3-one, 2— hydroxy — 9 — phenyl — phenalen — 75 — one, 3-hydroxy- oleanene core compounds. Among them, quercetin had the highest degree value and largest number of targets. Quercetin is a natural flavonoid compound that exists in the form of glycosides in fruits, herbs, and vegetables, after clinical trials, no obvious toxicity or side effects occurred, and it has various biological properties, such as anti-inflammatory, antioxidant, neuroprotective, and immunomodulatory properties.^[31–33]^ Its anti-inflammatory effect is mainly through inhibit the activation of NLRP3 inflammasome by inhibiting the production of cytokines, such as tumor necrosis factor (TNF)-α, interleukin (IL)-1β, and IL-6, and regulating Th17/Treg balance. thereby inhibiting the activation of NLRP3 inflammasome and reduces the inflammatory response of joints.^[34]^ The active ingredient can not only reduce the expression level of inflammatory cytokines but also promote the production of anti-inflammatory cytokines.^[32]^

## 5. Conclusions

Through network pharmacology and molecular docking, the potential targets and related pathways of Miao medicine Tongfengting decoction in the treatment of gout were studied. The results showed that a close relationship existed between the active compounds screened by Miao medicine Tongfengting Decoction and each target. Additionally, the active compounds had good binding activity. The study provides a novel idea of the mechanism of action of Miao medicine Tongfengting decoction in the treatment of gout. Additionally, it reflects the characteristics of a multi-target, multi-component, and multi-pathway strategy in the treatment of diseases by TCM, which is in line with the characteristics of TCM treatment based on dialectical treatment and overall concept.

## Author contributions

**Conceptualization:** Cong Huang.

**Data curation:** Xin Peng, Nannan Zhang, Yuepeng Cao.

**Investigation:** Zhigang Chen.

**Supervision:** Wukai Ma.

**Writing – original draft:** Xin Peng.

**Writing – review & editing:** Zhengqi Liu.

## Supplementary Material
