# Supplementary material for: The mechanism study of Miao medicine Tongfengting decoction in the treatment of gout based on network pharmacology and molecular docking
Source: Medicine (Baltimore). 2022 Dec 23;101(51):e32300. doi: 10.1097/MD.0000000000032300 (PMC9794283; doi:10.1097/MD.0000000000032300)
Supplement: Supplementary file 1 [file medi-101-e32300-s001.pdf]

Table1 Basic information of 183 active compounds from TCMSP

Chinese Herb	Mol ID	Molecule Name	OB	DL
Anemar rhena spp	MOL001677	asperglaucide	58.02	0.52
	MOL003773	Mangiferolic acid	36.16	0.84
	MOL000422	kaempferol	41.88	0.24
	MOL004373	Anhydroicaritin	45.41	0.44
	MOL004489	Anemarsaponin F_qt	60.06	0.79
	MOL004492	Chrysanthemaxanthin	38.72	0.58
	MOL004497	Hippeastrine	51.65	0.62
	MOL004514	Timosaponin B III_qt	35.26	0.87
	MOL000449	Stigmasterol	43.83	0.76
	MOL004528	Icariin I	41.58	0.61
	MOL004540	Anemarsaponin C_qt	35.5	0.87
	MOL004542	Anemarsaponin E_qt	30.67	0.86
	MOL000483	(Z)-3-(4-hydroxy-3-methoxy-phenyl)-N-[2-(4-hydroxyphenyl)ethyl]acrylamide	118.35	0.26
	MOL000546	diosgenin	80.88	0.81
	MOL000631	coumaroyltyramine	112.9	0.2
coix seed	MOL001323	Sitosterol alpha1	43.28	0.78
	MOL001494	Mandenol	42	0.19
	MOL002372	(6Z,10E,14E,18E)-2,6,10,15,19,23-hexamethyltetracosa-2,6,10,14,18,22-hexaene	33.55	0.42
	MOL002882	[(2R)-2,3-dihydroxypropyl] (Z)-octadec-9-enoate	34.13	0.3
	MOL000359	sitosterol	36.91	0.75
	MOL000449	Stigmasterol	43.83	0.76
	MOL008118	Coixenolide	32.4	0.43
	MOL008121	2-Monoolein	34.23	0.29
	MOL000953	CLR	37.87	0.68
砂仁	MOL001755	24-Ethylcholest-4-en-3-one	36.08	0.76
	MOL001771	poriferast-5-en-3beta-ol	36.91	0.75
	MOL001973	Sitosteryl acetate	40.39	0.85
	MOL000358	beta-sitosterol	36.91	0.75
	MOL003975	icosa-11,14,17-trienoic acid methyl ester	44.81	0.23
	MOL000449	Stigmasterol	43.83	0.76
	MOL007180	vitamin-e	32.29	0.7

	MOL007514	methyl icosa-11,14-dienoate	39.67	0.23
	MOL007535	(5S,8S,9S,10R,13R,14S,17R)-17-[(1R,4R)-4-ethyl-1,5-dimethylhexyl]-10,13-dimethyl-2,4,5,7,8,9,11,12,14,15,16,17-dodecahydro-1H-cyclopenta[a]phenanthrene-3,6-dione	33.12	0.79
	MOL007536	Stigmasta-5,22-dien-3-beta-yl acetate	46.44	0.86
Luoshiteng	MOL000359	sitosterol	36.91	0.75
	MOL000449	Stigmasterol	43.83	0.76
	MOL000522	arctiin	34.45	0.84
	MOL000525	Norwogonin	39.4	0.21
	MOL000527	luteolin-4'-O-β-D-glucoside	35.94	0.79
	MOL000528	voacangine	46.76	0.78
	MOL000529	Ibogain	50.52	0.63
	MOL000533	2(3H)-Furanone, 4-((3,4-dimethoxyphenyl)methyl)-3-((4-(beta-D-glucopyranosyloxy)-3-methoxyphenyl)methyl)dihydro-3-hydroxy-, (3S-cis)-	40.19	0.81
	MOL000006	luteolin	36.16	0.25
Phellodendri chinesis	MOL001454	berberine	36.86	0.78
	MOL001458	coptisine	30.67	0.86
	MOL002636	Kihadalactone A	34.21	0.82
	MOL013352	Obacunone	43.29	0.77
	MOL002641	Phellavin_qt	35.86	0.44
	MOL002643	delta 7-stigmastenol	37.42	0.75
	MOL002644	Phellopterin	40.19	0.28
	MOL002651	Dehydrotanshinone II A	43.76	0.4
	MOL002652	delta7-Dehydrosophoramine	54.45	0.25
	MOL002656	dihydroniloticin	36.43	0.81
	MOL002659	kihadanin A	31.6	0.7
	MOL002660	niloticin	41.41	0.82
	MOL002662	rutaecarpine	40.3	0.6
	MOL002663	Skimmianin	40.14	0.2
	MOL002666	Chelerythrine	34.18	0.78
	MOL000449	Stigmasterol	43.83	0.76
	MOL002668	Worenine	45.83	0.87
	MOL002670	Cavidine	35.64	0.81
	MOL002671	Candletoxin A	31.81	0.69
	MOL002672	Hericenone H	39	0.63
	MOL002673	Hispidone	36.18	0.83

	MOL000358	beta-sitosterol	36.91	0.75
	MOL000622	Magnograndiolide	63.71	0.19
	MOL000762	Palmidin A	35.36	0.65
	MOL000785	palmatine	64.6	0.65
	MOL000787	Fumarine	59.26	0.83
	MOL000790	Isocorypalmine	35.77	0.59
	MOL000098	quercetin	46.43	0.28
	MOL001131	phellamurin_qt	56.6	0.29
	MOL001455	(S)-Canadine	53.83	0.77
	MOL001771	poriferast-5-en-3beta-ol	36.91	0.75
	MOL002894	berberrubine	35.74	0.73
	MOL005438	campesterol	37.58	0.71
	MOL006392	dihydroniloticin	36.43	0.82
	MOL006401	melianone	40.53	0.78
	MOL006413	phellochin	35.41	0.82
	MOL006422	thalifendine	44.41	0.73
Radix Glycyrrhiza	MOL001484	Inermine	75.18	0.54
	MOL001792	DFV	32.76	0.18
	MOL000211	Mairin	55.38	0.78
	MOL002311	Glycyrol	90.78	0.67
	MOL000239	Jaranol	50.83	0.29
	MOL002565	Medicarpin	49.22	0.34
	MOL000354	isorhamnetin	49.6	0.31
	MOL000359	sitosterol	36.91	0.75
	MOL003656	Lupiwighteone	51.64	0.37
	MOL003896	7-Methoxy-2-methyl isoflavone	42.56	0.2
	MOL000392	formononetin	69.67	0.21
	MOL000417	Calycosin	47.75	0.24
	MOL000422	kaempferol	41.88	0.24
	MOL004328	naringenin	59.29	0.21
	MOL004805	(2S)-2-[4-hydroxy-3-(3-methylbut-2-enyl)phenyl]-8,8-dimethyl-2,3-dihydropyrano[2,3-f]chromen-4-one	31.79	0.72
	MOL004806	euchrenone	30.29	0.57
	MOL004808	glyasperin B	65.22	0.44
	MOL004810	glyasperin F	75.84	0.54
	MOL004811	Glyasperin C	45.56	0.4
	MOL004814	Isotrifoliol	31.94	0.42
	MOL004815	(E)-1-(2,4-dihydroxyphenyl)-3-(2,2-dimeth	39.62	0.35

		ylchromen-6-yl)prop-2-en-1-one		
	MOL004820	kanzonols W	50.48	0.52
	MOL004824	(2S)-6-(2,4-dihydroxyphenyl)-2-(2-hydroxypropan-2-yl)-4-methoxy-2,3-dihydrofuro[3,2-g]chromen-7-one	60.25	0.63
	MOL004827	Semilicoisoflavone B	48.78	0.55
	MOL004828	Glepidotin A	44.72	0.35
	MOL004829	Glepidotin B	64.46	0.34
	MOL004833	Phaseolinisoflavan	32.01	0.45
	MOL004835	Glypallichalcone	61.1	0.19
	MOL004838	8-(6-hydroxy-2-benzofuranyl)-2,2-dimethyl-5-chromenol	58.44	0.38
	MOL004841	Licochalcone B	76.76	0.19
	MOL004848	licochalcone G	49.25	0.32
	MOL004849	3-(2,4-dihydroxyphenyl)-8-(1,1-dimethylprop-2-enyl)-7-hydroxy-5-methoxy-coumarin	59.62	0.43
	MOL004855	Licoricone	63.58	0.47
	MOL004856	Gancaonin A	51.08	0.4
	MOL004857	Gancaonin B	48.79	0.45
	MOL004860	licorice glycoside E	32.89	0.27
	MOL004863	3-(3,4-dihydroxyphenyl)-5,7-dihydroxy-8-(3-methylbut-2-enyl)chromone	66.37	0.41
	MOL004864	5,7-dihydroxy-3-(4-methoxyphenyl)-8-(3-methylbut-2-enyl)chromone	30.49	0.41
	MOL004866	2-(3,4-dihydroxyphenyl)-5,7-dihydroxy-6-(3-methylbut-2-enyl)chromone	44.15	0.41
	MOL004879	Glycyrin	52.61	0.47
	MOL004882	Licocoumarone	33.21	0.36
	MOL004883	Licoisoflavone	41.61	0.42
	MOL004884	Licoisoflavone B	38.93	0.55
	MOL004885	licoisoflavanone	52.47	0.54
	MOL004891	shinpterocarpin	80.3	0.73
	MOL004898	(E)-3-[3,4-dihydroxy-5-(3-methylbut-2-enyl)phenyl]-1-(2,4-dihydroxyphenyl)prop-2-en-1-one	46.27	0.31
	MOL004903	liquiritin	65.69	0.74
	MOL004904	licopyranocoumarin	80.36	0.65
	MOL004905	3,22-Dihydroxy-11-oxo-delta(12)-oleanene-27-alpha-methoxycarbonyl-29-oic acid	34.32	0.55
	MOL004907	Glyzaglabrin	61.07	0.35

MOL004908	Glabridin	53.25	0.47
MOL004910	Glabranin	52.9	0.31
MOL004911	Glabrene	46.27	0.44
MOL004912	Glabrone	52.51	0.5
MOL004913	1,3-dihydroxy-9-methoxy-6-benzofurano[3,2-c]chromenone	48.14	0.43
MOL004914	1,3-dihydroxy-8,9-dimethoxy-6-benzofurano[3,2-c]chromenone	62.9	0.53
MOL004915	Eurycarpin A	43.28	0.37
MOL004917	glycyroside	37.25	0.79
MOL004924	(-)-Medicocarpin	40.99	0.95
MOL004935	Sigmoidin-B	34.88	0.41
MOL004941	(2R)-7-hydroxy-2-(4-hydroxyphenyl)chroman-4-one	71.12	0.18
MOL004945	(2S)-7-hydroxy-2-(4-hydroxyphenyl)-8-(3-methylbut-2-enyl)chroman-4-one	36.57	0.32
MOL004948	Isoglycyrol	44.7	0.84
MOL004949	Isolicoflavonol	45.17	0.42
MOL004957	HMO	38.37	0.21
MOL004959	1-Methoxyphaseollidin	69.98	0.64
MOL004961	Quercetin der.	46.45	0.33
MOL004966	3'-Hydroxy-4'-O-Methylglabridin	43.71	0.57
MOL000497	licochalcone a	40.79	0.29
MOL004974	3'-Methoxyglabridin	46.16	0.57
MOL004978	2-[(3R)-8,8-dimethyl-3,4-dihydro-2H-pyrano[6,5-f]chromen-3-yl]-5-methoxyphenol	36.21	0.52
MOL004980	Inflacoumarin A	39.71	0.33
MOL004985	icos-5-enoic acid	30.7	0.2
MOL004988	Kanzonol F	32.47	0.89
MOL004989	6-prenylated eriodictyol	39.22	0.41
MOL004990	7,2',4'-trihydroxy － 5-methoxy-3 － arylcoumarin	83.71	0.27
MOL004991	7-Acetoxy-2-methylisoflavone	38.92	0.26
MOL004993	8-prenylated eriodictyol	53.79	0.4
MOL004996	gadelaidic acid	30.7	0.2
MOL000500	Vestitol	74.66	0.21
MOL005000	Gancaonin G	60.44	0.39
MOL005001	Gancaonin H	50.1	0.78
MOL005003	Licoagrocarpin	58.81	0.58

	MOL005007	Glyasperins M	72.67	0.59
	MOL005008	Glycyrrhiza flavonol A	41.28	0.6
	MOL005012	Licoagroisoflavone	57.28	0.49
	MOL005013	18α-hydroxyglycyrrhetic acid	41.16	0.71
	MOL005016	Odoratin	49.95	0.3
	MOL005017	Phaseol	78.77	0.58
	MOL005018	Xambioona	54.85	0.87
	MOL005020	dehydroglyasperins C	53.82	0.37
	MOL000098	quercetin	46.43	0.28
Sargentodoxae caulis	MOL000358	beta-sitosterol	36.91	0.75
	MOL000359	sitosterol	36.91	0.75
	MOL007920	meso-1,4-Bis-(4-hydroxy-3-methoxyphenyl)-2,3-dimethylbutane	31.32	0.26
	MOL007923	2-(4-hydroxyphenyl)ethyl (E)-3-(4-hydroxyphenyl)prop-2-enoate	93.36	0.21
	MOL000096	(-)-catechin	49.68	0.24
chuanniuxi	MOL012286	Betavulgarin	68.75	0.39
	MOL012298	Rubrosterone	32.69	0.47
	MOL000358	beta-sitosterol	36.91	0.75
	MOL000098	quercetin	46.43	0.28
smilax tuber	MOL013233	EINECS 213-897-0	71.96	0.72
	MOL000546	diosgenin	80.88	0.81
